# Metal carcinogens and cancer: integrating genetic and epigenetic perspectives

**DOI:** 10.3389/fonc.2026.1896466

**Published:** 2026-08-12

**Authors:** Shipra Singh, Priyanka Phogat, Luciano Saso, Shrikant Kukreti, Aparna Bansal

**Affiliations:** 1 Nucleic Acids Research Lab, Department of Chemistry, University of Delhi, Delhi, India; 2 Department of Chemistry, Hansraj College, University of Delhi, Delhi, India; 3 Department of Physiology and Pharmacology “Vittorio Erspamer”, Sapienza University of Rome, Rome, Italy

**Keywords:** cancer, carcinogenesis, epigenetic modification, genetic mutation, toxic metals

## Abstract

People around the globe are affected by extensive environmental and occupational exposure to heavy metals, which poses a significant threat to various health hazards, with cancer being a major concern. These toxic metals are ubiquitously found in food, air, water, and industrial, agricultural, or pharmaceutical applications. Being non-biodegradable, they accumulate in the living organism. The prolong exposure to these metals can result into hazardous effects in the many organs including respiratory problems, gastrointestinal disorders, renal issues, and skin lesions etc. Arsenic (As), Cadmium (Cd), Chromium (Cr), Nickel (Ni), Mercury (Hg), and Lead (Pb) are the most common heavy metals causing carcinogenicity through both genetic and epigenetic mechanisms. The literature indicates that DNA damage, deregulation of gene expression, interference with cell signaling, etc., contribute to metal-induced carcinogenesis. Changes in epigenetic processes, such as DNA methylation, histone modification, chromatin remodeling, and non-coding RNAs, can disturb normal cell function and may even lead to cancer. However, the molecular mechanism remains poorly understood. Advancements in the field have underscored the critical roles of genetic mutations and epigenetic modifications in metal-induced tumor formation. This review gives a comprehensive overview of the function of metals in causing cancer, focusing on pathways such as genotoxicity, mutagenesis, epigenetic modification, and cancer-related signaling. Furthermore, with the ultimate goal of reducing the burden of this disease, the targeted preventive strategies, such as reducing the emission of these toxic metals into the environment from mining to industries, or other sources, and the need for social interventions, have been discussed.

## 1 Introduction

Metals are essential constituents of the earth’s crust, and a lot of them are required for life because they regulate physiological and metabolic processes (1). Carcinogenesis, the intricate process of cancer development, is the result of complex interactions between genetic mutations and environmental factors, with heavy metals recognized as important initiators and promoters (2). Heavy metal contamination in the atmosphere includes air, water and soil, and is mostly caused by human activities such as fossil fuel burning, automotive emissions, waste incineration, and industrial operations, including mining and agriculture (3, 4). Metal pollution in the environment has been a serious issue for both nature and public health around the world, because metals can be hazardous for people and other organisms (5). Prolonged exposure to carcinogenic metals such as As, Cd, Cr, Ni, and Be is a significant yet underrecognized factor contributing to cancer risk globally. Hundreds of millions of people are affected by these metals through environmental or occupational exposure. These metals promote carcinogenesis *via* intricate genetic and epigenetic mechanisms that disturb cellular homeostasis and gene regulation, in contrast to conventional mutagens. Epidemiological and experimental studies have demonstrated that metal poisoning can impair DNA repair mechanisms, induce oxidative damage, and alter signal transduction pathways, thereby directly contributing to genomic instability and the malignant transformation of cells (6–8). Reactive oxygen species (ROS) generate oxidative stress, a well-known mechanism by which heavy metals can harm cells. Heavy metals are used in a lot of industrial items, even though they are quite poisonous. For example, they are in batteries, paints, and car exhaust (9). Lead (Pb) and other metals present in paint have caused poisoning in children who ingest paint chips containing Pb, as well as in adults engaged in renovating old furniture and dwellings coated with Pb-based paint. We continue to produce lead crystal for wine glasses and decanters, which results in lead leaching from the container into the wine consumed. Likewise, we utilize silver amalgam restorations to restore decayed teeth; these fillings contain approximately 50% mercury by weight (10, 11).

Arsenic and nickel are two metals that cause worldwide DNA hypomethylation, which disrupts the genome, rearranges chromosomes, and leads to spontaneous mutations. At the same time, they induce promoter-specific hypermethylation, which inhibits tumor suppressor genes. According to research, arsenic causes dose-dependent hypomethylation that persists after exposure, followed by hypermethylation at loci such as p53 promoters, which correlates with a prolonged carcinogenic potential (1, 12–14). Nickel also raises genome methylation levels, which correlates with heterochromatinization and the inhibition of genes such *MAP2K3* and *DKK1* (15).

**Figure 1:**
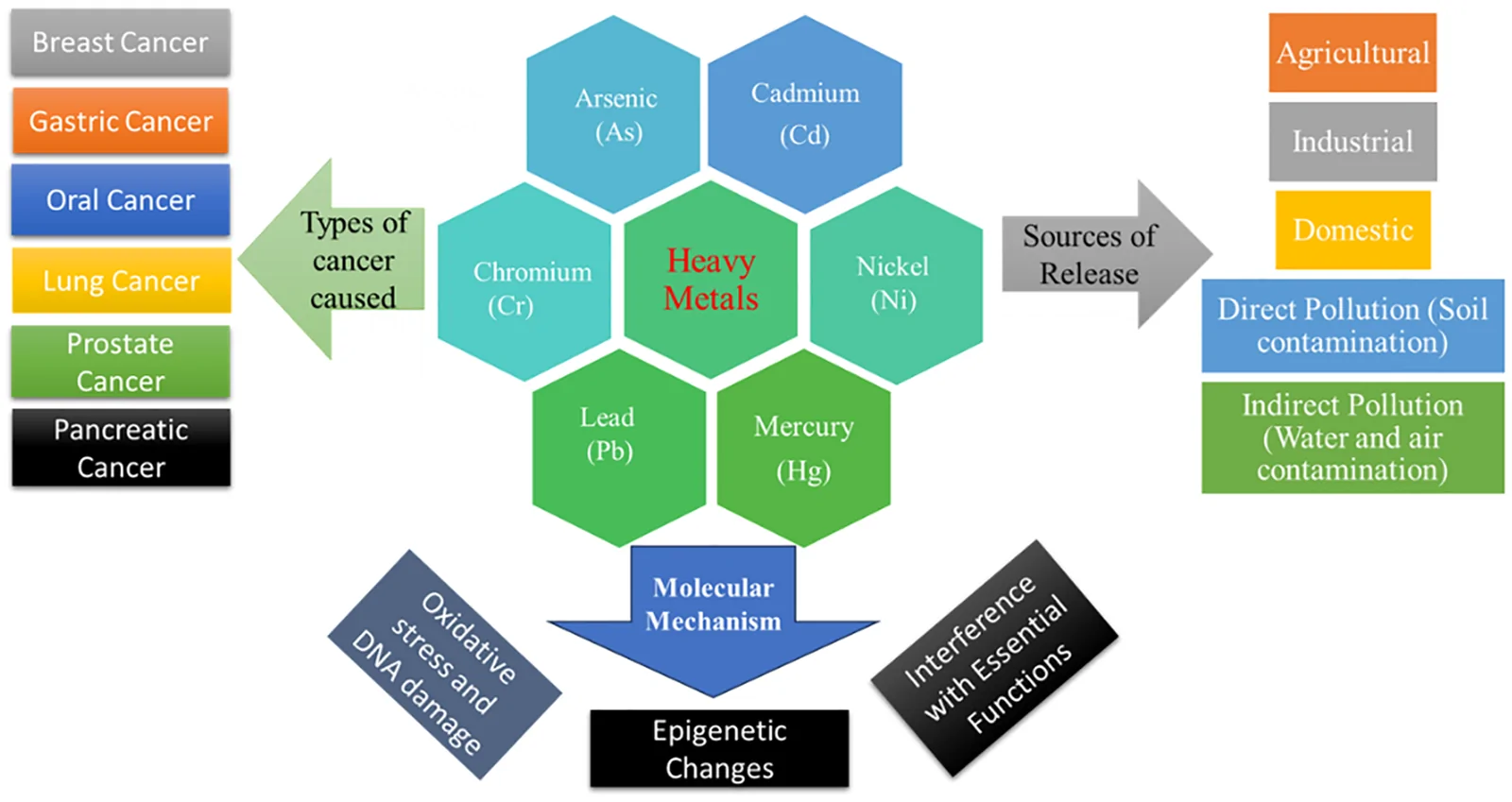
Sources and adverse effect of heavy metals.

Pollution from metals is a global concern. They are persistent environmental pollutants because microorganisms do not break them down like they do organic pollutants. Therefore, they build up and bioconcentrate in our ecosystems (6). Over the past few decades, industry, agriculture, pharmaceuticals, and technology have all seen a tremendous rise in the use of metals (7). The International Agency for Research on Cancer (IARC) categorizes several of these metals, along with their compounds, within Group 1, denoting established human carcinogens, which means they are known to cause cancer in many organ systems, such as the lung, skin, liver, bladder, and kidney (2). *In vitro* transformation assays together with *in vivo* animal investigations offer persuasive evidence of carcinogenicity, capturing both the malignant conversion of normal cells and tumor induction in animals. The processes implicated in heavy metal-driven carcinogenesis include oxidative stress, alterations in DNA damage and repair, and disturbances in signal transduction pathways (16) (**Figure 1**).

Epigenetics is the study of dynamic, heritable changes in chromatin that do not include changes in the genomic sequence. Epigenetic regulation is an important step in controlling gene expression (17). The recent advances in the knowledge of epigenetics have revealed the significance of epigenetic changes in the genesis of cancer. Epigenetic modifications represent biologically meaningful changes capable of influencing gene expression without any change to the DNA sequence itself (14). Histone modification and non-coding RNA (ncRNA) constitute two key epigenetic mechanisms and, in conjunction with DNA methylation, are implicated in gene silencing. Such processes are important for gene expression regulation and cell growth (18). Beyond conventional genotoxic effects, recent advances have established epigenetic pathways as important players in metal-induced carcinogenesis. Metals affect histone modifications, DNA methylation and non-coding RNA networks to maintain oncogenic states (19). This review focuses on the current understanding of metal-induced carcinogenesis, with a particular emphasis on the genetic and epigenetic pathways that promote cancer growth.

## 2 Biomapping of toxic metals

The biomapping of toxic metals has become a convincing method to detect them in normal human cells where common cancers originate. Pamphlett and Bishop have nicely shown biomapping of toxic elements prone to cancer development using autometallography and laser therapy inductively coupled-mass spectrometry imaging methods. With the help of these techniques, it became possible not only to explain the presence of metal toxicants causing cancer but also the increasing incidence of cancers with ageing (20). It has been observed that the human cells that harbour the toxic metals most often are breast, ovary, kidney, adrenal gland, anterior pituitary, liver, nervous system and endothelial cells in many organs etc. (**Table 1**).

**Table 1:** Brief overview of metal toxicity in human body.

S.no	Organ	Toxic metal	Disease	Ref
1.	Kidney	Mercury (major) mixture of cadmium, lead, nickel and silver also present	Clear cell carcinoma (renal cancer)	(20)
2.	Breast	Mercury (major), other metals like nickel, iron, aluminum, chromium and cadmium also present	Breast cancer	(21)
3.	Pancreas	Mercury (major) cadmium, chromium, lead, nickel also detected	pancreatic cancer	(22)
4.	liver	Mercury	hepatocellular carcinoma, biliary carcinoma	(23)
5.	Anterior pituitary	Mercury	Growth hormone-secreting somatotroph adenomas	(24)
6.	Adrenal gland	Mercury	adrenal adenoma	(25)

Today, Cancer has become one of the most prevalent non-communicable diseases globally. Around 20 million cases and 9 million deaths were reported in 2022 which is estimated to grow to 35 million by 2050 (26). Among various cancer-causing factors, metal carcinogenicity is one of the well-discussed topics in toxicology. According to IARC, As, Cd, Cr, Ni and their compounds are classified as group 1 carcinogens (7).

**Figure 2:**
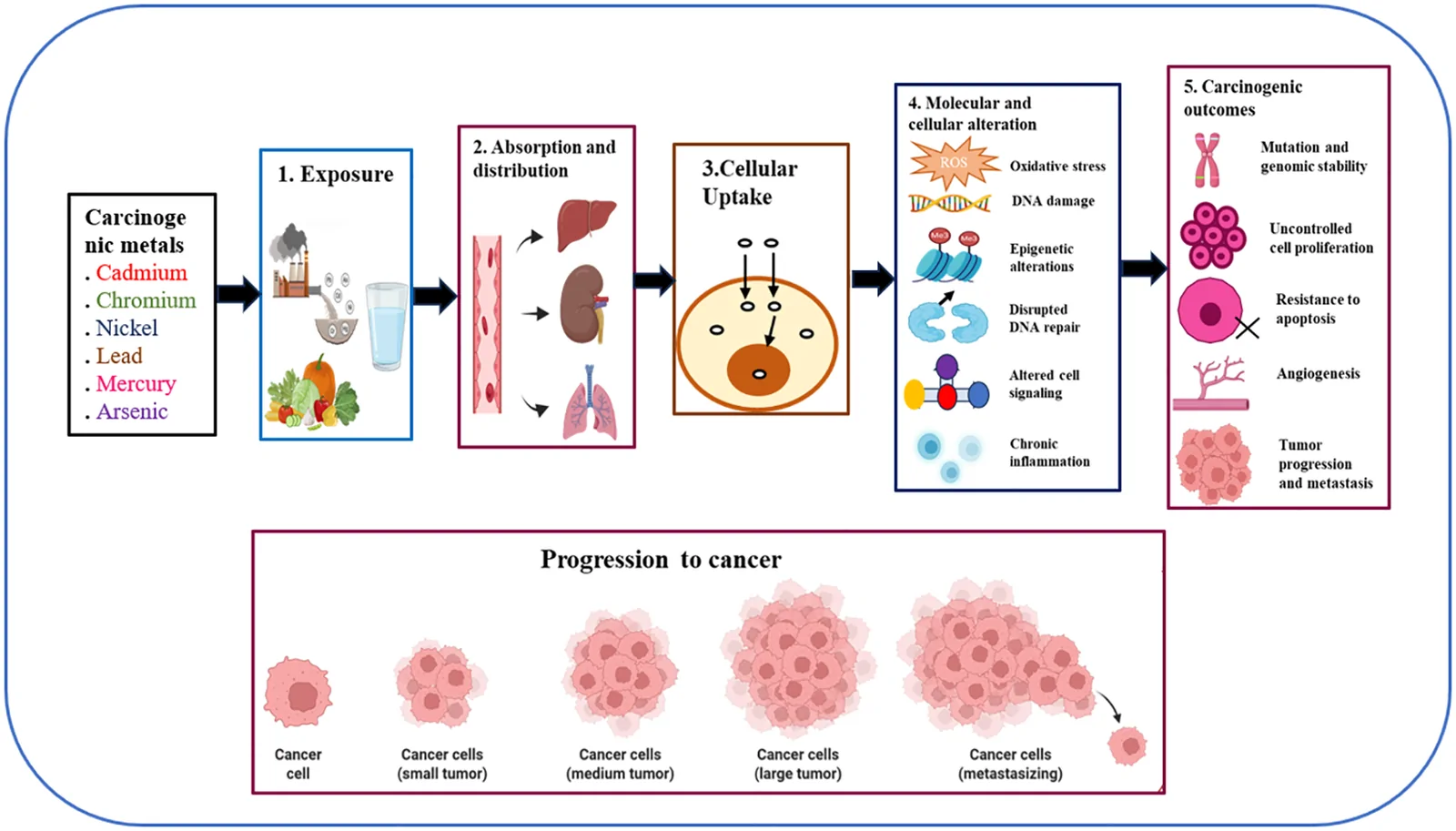
Schematic representation of metal carcinogenesis.

Chronic exposure to metals and their compounds is capable of inducing cancer in both humans and animals, acting through organelle damage, disturbance of redox homeostasis within the cell, dysregulation of cell-cycle control and development, and disruption of DNA repair processes etc. (27). A comprehensive summary of the actions of these metal carcinogens has been discussed, focusing on various pathways like genotoxicity, mutagenesis, oxidative stress and epigenetic modifications, etc. (**Figure 2**).

### 2.1 Cadmium

Cadmium is a highly poisonous, non-essential transition element that poses a serious health hazard to both humans and animals. It occurs naturally in the environment and is released into the environment as a pollutant from various agricultural and industrial activities (28). In nature, cadmium is typically found in association with ores of zinc, lead, and copper within the Earth’s crust (29). Natural processes such as weathering of rocks and volcanic eruptions, contribute to its environmental presence; however, human activities remain the primary source of cadmium exposure (30, 31). Major anthropogenic sources include smelting and mining of non-ferrous metals, manufacturing of nickel-cadmium batteries, electroplating operations, pigment production, plastic stabilizers, fossil fuel combustion, and waste incineration processes (32). Increasing industrialization, particularly in developing nations, has further intensified cadmium contamination in the environment. Cadmium’s prolonged biological half-life, estimated at 10 to 30 years, contributes to its progressive bioaccumulation in tissues, thereby increasing its toxic potential (30). Human exposure primarily occurs through food consumption, tobacco smoke, occupational settings, and contaminated drinking water. Among these, cigarette smoke represents a major exposure route, as tobacco plants efficiently absorb cadmium from contaminated soils (28, 33). Due to strong epidemiological evidence linking cadmium exposure with cancers of the lung, kidney, and prostate, cadmium and its compounds have been classified as Group 1 human carcinogens by the IARC (34).

#### 2.1.1 Cadmium toxicity

Cadmium toxicity is primarily driven by redox imbalance, inflammatory responses, and disruption of fundamental cellular homeostasis. Although cadmium is not a redox-active metal and does not directly generate ROS and hence indirectly cause oxidative stress by depleting intracellular glutathione pools and disrupting important antioxidant defense enzymes such as, glutathione peroxidase, catalase and superoxide dismutase (35, 36). Imbalance of cellular redox status causes oxidative harm to macromolecules, and is one of the major factors responsible for cadmium-induced cytotoxicity.

Chronic cadmium exposure has been associated with multi-organ toxicity affecting the kidneys, liver, lungs, skeletal system, and reproductive organs. The kidney represents the most critical target organ due to preferential congestion of cadmium in proximal tubular epithelial cells leading to tubular degeneration; proteinuria; decreased glomerular filtration efficiency (30, 37). At the molecular level, cadmium activates several oncogenic signaling cascades, including NF-κB, PI3K/AKT, MAPK, and Wnt/β-catenin pathways (31). Dysregulation of these pathways promotes chronic inflammation, suppresses apoptotic signaling, and enhances uncontrolled cellular proliferation, thereby facilitating tumor initiation and progression (38). Moreover, cadmium has been shown to impair mitochondrial bioenergetics, disrupt intracellular calcium homeostasis, and reprogram cellular metabolism, thereby further amplifying its toxic and carcinogenic potential.

#### 2.1.2 Epigenetic effects of cadmium toxicity

Cd exposure exerts substantial epigenetic effects by modulating DNA methylation patterns, histone post-translational modifications, and ncRNA expression, thereby producing heritable alterations that may contribute to transgenerational toxicity. Prenatal cadmium exposure has been shown to induce hypermethylation at the *H19* imprint control region and reduce histone modifications, including H3K4me2 and H4K12ac, in offspring oocytes. These epigenetic changes negatively affect meiotic maturation, early embryonic development, and ovarian gene networks associated with metabolic regulation and cellular signaling (39, 40).

Cadmium exposure in somatic cells induces dynamic changes in DNA methylation, initially causing hypomethylation through DNMT inhibition and subsequently promoting chronic global hypermethylation that leads to epigenetic repression of tumor suppressor genes, including *p16INK4a*, and DNA repair mechanisms. In the placenta, cadmium exposure has been linked with promoter hypomethylation of *Cdkn1c*, potentially enhancing growth signals, whereas hypermethylation of *Peg10* is associated with impaired fetal development. Furthermore, cadmium inhibits histone demethylase activity, leading to the accumulation of histone modifications, including H3K4me3 and H3K9me2. Cadmium also modulates non-coding RNA networks, in which increased miR-21 expression suppresses PDCD4-mediated apoptosis, while altered lncRNA expression promotes stemness characteristics and kidney toxicity. These epigenetic alterations have been shown to persist across generations in both murine models and human populations, highlighting their role in Cd-induced reproductive disorders, cancer development, and ageing-related pathologies (40–45).

### 2.2 Chromium

Cr is a transition metal (atomic number 24) that naturally occurs in mineral ores such as ferric chromite (FeCr_2_O_4_), crocoite (PbCrO_4_), and chromium oxide (Cr_2_O_3_). It is categorized as one of the most widely distributed elements in the crust of the earth and it is the 6^th^ most common transition metal (46, 47). It has gained significant attention due to its environmental and occupational health implications and has been classified as a human cancer-causing agent by the National Toxicology Program (48). It is a naturally sourced heavy metal extensively used in industrial activities and may lead to serious health effects in humans (49).

Chromium is present in the environment mainly in two valence states, i.e., Cr (VI) and Cr (III). While hexavalent chromium, Cr (VI), finds application in many industrial processes, trivalent chromium, Cr (III), can function as a micronutrient and nutritional supplement. The toxicity of Cr (III) compounds is thought to be on the order of 100-fold lower than that of Cr (VI) (50, 51). Hexavalent Cr (VI) is a highly soluble, oxidizing species and it is formed through both natural and anthropogenic processes (52). In the manufacturing procedures of the salt form of the trivalent and hexavalent Cr compounds called chromate, the hazardous dust is generated (53). Cr dust has been extensively studied for its negative impact on workers producing chromate. Chromium-containing industrial waste is a notable source of pollution in soil and water systems and, as such, may exert serious effects on human health (54–56). Contributing sources that elevate ambient chromium include mining operations, the steel and metal-alloy sectors, paint production, wood and paper processing, coal-ash combustion, municipal-waste-to-energy use, and second-generation fertilizer production (57). Chromium exists in contaminated drinking water (58) and its effects other than oncological include an endocrine disruptive potential (59).

#### 2.2.1 Chromium toxicity

The toxicological effects of chromium are primarily determined by its valence state and the pathway of exposure. Hexavalent chromium [Cr (VI)] demonstrates significantly greater toxicity than trivalent chromium [Cr (III)] due to its efficient cellular uptake through anion transport systems such as sulfate channels (60). Following intracellular entry, Cr (VI) undergoes sequential reduction to lower oxidation states, forming reactive chromium species and ROS, which are key mediators of chromium-induced cytotoxicity (61). Excessive ROS production leads to oxidative damage to essential biomolecules, including nucleic acids, proteins, and membrane lipids. This redox imbalance further contributes to mitochondrial dysfunction, inhibition of enzymatic activities, and loss of membrane integrity. Consistent with these findings, several studies have reported elevated levels of oxidative stress biomarkers, along with suppression of cellular antioxidant defense systems, following chromium exposure (62, 63).

Chromium toxicity is also associated with inflammatory and immune responses. Upon chromium exposure, central signaling routes including NF-κB and MAPK are activated, accompanied by enhanced release of pro-inflammatory cytokines such as TNF-α and IL-6 (64). Persistent inflammation has the potential to drive tumorigenesis by encouraging cell division and limiting apoptosis (65).

#### 2.2.2 Epigenetic regulation in chromium carcinogenesis

Epigenetic changes are increasingly recognized as important aspects of chromium-induced carcinogenesis. Exposure to chromium is capable of modifying DNA methylation profiles, histone marks, and non-coding RNA control, effects that shape gene expression while leaving the DNA sequence itself intact (66).

Cr (VI) exposure is known to cause hypermethylation in the promoter regions of genes involved in tumor suppression and DNA repair and thus reduce their expression. It may also affect the histone methylation, leading to chromatin structural changes that repress gene expression. Additionally, chromium may alter microRNA expression patterns associated with oxidative damage and oncogenic signaling, suggesting their potential utility as exposure indicators (67).

### 2.3 Lead

Lead (Pb) is a heavy metal with atomic number 82 that occurs naturally in low concentrations (10–20 ppm) in the Earth’s crust, primarily as galena (PbS). However, human activities have mined and dispersed it across the country, causing significant environmental damage, including contamination of the soil and water resources, which is a severe health threat to both humans and wildlife. Lead is the most toxic heavy metal in the environment. Because of its significant physical and chemical properties, it has been employed since ancient times. It is a widely distributed, important, but hazardous substance in the global ecosystem (68, 69).

Lead is profoundly harmful to people, animals, and ecosystems alike. It builds up in the skeleton, liver, and kidneys, rendering it particularly injurious to children, adolescents, and expectant mothers (68). The presence of lead in drinking water has been recognized over many years as a major exposure pathway. Further sources include candies, folk and traditional treatments, glazed ceramic ware, toys and jewelry intended for children, clothing embellishments, key rings and other metallic or painted objects, and goods derived from vinyl, plastic, and rubber. Following the phase-out of leaded gasoline, paints high in lead have become the dominant exposure source for populations worldwide (70, 71). Individuals working in lead-handling industries, such as battery and vehicle manufacturing, refining, and smelting operations, encounter lead and its compounds occupationally. Lead interferes with several biological processes and is toxic to the heart, kidneys, gastrointestinal tract and neurological system; the latter being the most vulnerable. Lead also interferes with children’s brain development, leading to cognitive issues (71, 72). In many instances, metallic lead does not respond well to certain environmental or biological conditions (73). When heated or exposed to damp air, it can oxidize and produce a variety of inorganic and organic lead compounds, which can then degrade into other lead materials, such as organolead and alkyl lead (73). Inorganic lead is most commonly encountered in dust, soil, weathered paint, and assorted consumer goods, in contrast to organic lead (tetraethyl lead), which is found primarily in leaded fuel. Toxicity characterizes both forms, yet organic lead complexes are substantially more damaging to living systems than the inorganic form (74), owing to their capacity to enter biological systems with ease and disturb cellular processes, thereby causing serious health consequences.

#### 2.3.1 Lead toxicity

Environmental scientists are interested in Pb toxicity because it is detrimental to plants, animals, and humans (74). Lead is extremely toxic and can harm the brain, body, and mind in several ways. The worldwide threshold for lead poisoning is 10 μg/dL in blood (75, 76).

Lead toxicity is a major concern for public health, as it has the potential to affect multiple organ systems even at low exposure levels. Lead adheres to red blood cells after inhalation or ingestion, then travels to soft tissues such as the liver, kidneys, and brain. It is then retained in bones, where it might remain for decades (77, 78). Lead toxicity at the molecular level is primarily caused by oxidative stress, disruption of antioxidant defense mechanisms, and interference with essential metal ions such as Ca, Fe, and Zn. This condition reduces enzyme efficiency and disrupts cellular signaling pathways (79, 80). Lead interferes with neurotransmitter release, synaptic plasticity, and neuronal development in the nervous system, increasing the likelihood of cognitive impairments, behavioral issues, and lower IQs (81). Lead exposure has also been associated with haematological repercussions, such as heme production inhibition, which causes anemia, renal failure, and cardiovascular abnormalities (27). New research indicates that alterations in histone modifications and DNA methylation are critical for the long-term, detrimental, and perhaps cancer-causing effects of lead (82). It is vital to stress that there is no safe amount of lead exposure, emphasizing the importance of continuous monitoring and preventive steps to mitigate its health impacts (80).

#### 2.3.2 Epigenetic regulation in lead carcinogenesis

Lead (Pb) is a possible human carcinogen (IARC Group 2A), influencing carcinogenesis through epigenetic processes rather than direct genotoxicity, with dose- and tissue-specific changes emerging at low blood lead levels (BLLs) < 40 µg/dL (83, 84). Occupational cohorts exhibit differentially methylated positions (DMPs) in cell cycle genes; specifically, *RRAGC* and *UBE2V1* show hypomethylation inversely linked to BLLs, mediating approximately 19% of genotoxicity through G2/M checkpoint dysregulation and comet-detected DNA damage. Overexpression of miR-148a directly inhibits DNMT1, leading to LINE-1 hypomethylation and promoting genomic instability and potential oncogenesis (85, 86). Prenatal Pb causes hypomethylation at particular loci in human embryonic stem cells (hESCs), which messes up neuronal development and global methylation patterns. Rodent models demonstrate promoter hypermethylation of tumor suppressors, accompanied by transgenerational oocyte alterations, specifically, a reduction in H3K4me2 and hypermethylation of H19 ICR, which persist throughout the F2/F3 generations. Histone effects encompass H3/H4 acetylation deficits in neuronal cells, H3K4me2 elevation in primate brains following developmental exposure, and Pb-Zn competition that disturbs the HDAC/HAT equilibrium essential for DNA repair through H2A ubiquitination; Pb replaces Zn in protamine transcriptional regulators, diminishing DNA binding and epigenetic regulation (87–91).

MiRNA dysregulation can lead to leukemia and kidney malignancies by inducing miR-155 (NF-κB/inflammation) and downregulating miR-126. Pb also inhibits chromatin accessibility via ROS synergy (18). Despite the potential of biomarkers (RRAGC methylation), tumor-specific validation is delayed, with Pb acting as a co-promoter in multi-metal settings (Cd-Cr-Pb) (92), complicating understanding of the interactions among these metals and their collective impact on cancer progression.

### 2.4 Arsenic

Arsenic (As), a naturally occurring hazardous metalloid with an atomic number of 33, poses significant health risks when combined with drinking water, mostly leading to various forms of cancer in humans (e.g., skin, liver, lung, bladder, and prostate) (93). Ubiquitously present in the environment and extensively utilized in mining, smelting, insecticides, wood preservatives, pigments, and the semiconductor industry (94). Various sectors, including wood preservatives, textiles, glass, and cosmetics, also contribute to arsenic poisoning (95). Poisoning can impair the immune system and trigger inflammatory processes. Multiple investigations have elucidated the carcinogenic, mutagenic, and genotoxic mechanisms of arsenic (96).

Arsenic can adapt various oxidation state (0, + 3, +5) and exists in various forms like organic, and arsine (AsH_3_), each exhibiting distinct levels of toxicity (97). Inorganic arsenic compounds are highly toxic and carcinogenic, causing skin lesions, cardiovascular disorders, neurotoxicity, hepatotoxicity, nephrotoxicity, and malignancies in the skin, lung, bladder, liver, and kidney (98). By impairing key DNA repair mechanisms such as nucleotide excision repair, base excision repair, and mismatch repair, it induces genotoxicity and drives mutations in tumor suppressor genes, including p53 (99, 100). As has been shown to affect chromosomal instability in several previous investigations (101). Moreover, it leads to the generation of numerous ROS, like peroxyl, hydroxyl, dimethyl arsenic radicals, and hydrogen peroxide, which thus cause oxidative DNA destruction (102).

It can infiltrate the biosphere via dust, rainwater, groundwater, or human activities, alongside natural sources such as volcanic eruptions, the weathering of arsenic-laden rocks, and geothermal processes, as well as anthropogenic actions including mining, coal combustion, pesticide application, and industrial waste discharge that contaminate the environment. A quantity exceeding 10 µg/L in drinking water or soil is concerning for human health (80, 103). Drinking groundwater contaminated with arsenic is a sure way to get poisoned, mainly in India, China, Bangladesh, and some Central and South American countries. Extensive study has been conducted on the toxicological effects of arsenic in various animal models to elucidate the underlying mechanisms of arsenic toxicity (104). At the molecular level, arsenic induces oxidative stress, mitochondrial dysfunction, DNA damage, poor DNA repair and epigenetic changes leading to toxicity and cancer (105). IARC categorizes inorganic arsenic compounds as Group 1 human carcinogens (106). The World Health Organization (WHO) reported that high arsenic exposure in humans causes chronic disorders (93).

#### 2.4.1 Arsenic toxicity

Oxidative stress and disruption of cellular metabolism primarily contribute to arsenic’s toxicity. Arsenic promotes the overproduction of reactive ROS, protein oxidation, leading to lipid peroxidation, oxidative DNA damage and mitochondrial impairment (105). ROS induce damage that compromises membrane integrity and facilitates chromosomal abnormalities, DNA strand breakage, and genomic instability. Arsenic binds to sulfhydryl (-SH) groups in proteins and enzymes, obstructing essential metabolic pathways, including cellular respiration, ATP synthesis, and antioxidant defense mechanisms (107). Moreover, arsenic disrupts vital trace elements such as selenium and zinc, thereby hindering proper cellular homeostasis and immunological function.

Acute high-dose ingestion (>5 mg/kg, e.g., contaminated water/pesticides) results in acute diarrhoea, hypovolemia, hepatic failure, QT prolongation, and encephalopathy, with a mortality rate of 10-30% despite chelation (BAL, succimer) (103). Chronic low-dose exposure (10-500 μg/L groundwater) induces multi-organ effects: hyperpigmentation/hypopigmentation, hyperkeratosis, peripheral neuropathy, Blackfoot disease (vasculopathy), diabetes (disruption of insulin signaling), and hypertension. As a Group 1 IARC carcinogen, arsenic produces squamous cell carcinoma of the skin, as well as lung, bladder, liver, and kidney malignancies through iAs(III)-DNA adducts, chromosomal abnormalities, and global DNA hypomethylation, which involves DNMT1/3A suppression and SAM depletion, hence facilitating oncogene activation, particularly *KRAS* (107–110). Arsenic, due to its widespread environmental contamination and multi-organ toxicity, poses a significant environmental and occupational risk that requires ongoing surveillance and the development of effective preventive and therapeutic measures.

#### 2.4.2 Epigenetic mechanisms in arsenic carcinogenesis

Epigenetic control significantly alters gene expression during arsenic carcinogenesis without altering DNA sequences (111). Chronic arsenic exposure induces several epigenetic alterations, including abnormalities in histone modifications, DNA methylation, and disruptions of ncRNAs, all of which facilitate tumor growth (112, 113). A major mechanism is the depletion of S-adenosylmethionine (SAM), which results in global DNA hypomethylation and promoter-specific hypermethylation of tumor suppressor genes, ultimately causing genomic instability and transcriptional silencing (114, 115). Arsenic-induced histone modifications lead to increased repressive marks such as H3K9me2 and H3K27me3 along with reduced histone acetylation, thereby suppressing apoptosis and DNA repair pathways (116).

Moreover, arsenic generates ROS, which activate NF-κB and MAPK signaling pathways, further enhancing epigenetic dysregulation and compromising genomic integrity (103). Chronic arsenic exposure also disrupts the miRNAs and lncRNAs regulation, thereby promoting oncogenic signaling, angiogenesis, and cancer progression (117). These cumulative epigenetic alterations are strongly associated with arsenic-induced cancers of the skin, lung, bladder, liver, and kidney, highlighting the importance of further research into biomarkers and targeted therapeutic strategies for arsenic-related malignancies (107, 118).

### 2.5 Nickel

It is a hard, ductile, silver-white transition metal (atomic number = 28) of the periodic table. Ni^2+^ is common oxidation state in the environment and biological systems (16). Nickel exposure is mostly caused by its use in industries such as stainless steel and battery production, as well as contamination of food and water. These have been linked to different health hazards, including cardiovascular and kidney problems, dermatitis, and carcinogens (119). According to IARC, metallic nickel is a group 2B human carcinogen, whereas nickel compounds are group 1 carcinogens (120).

Millions of workers worldwide are typically at risk of occupational exposure in these industrial settings (121). Depending on its chemical speciation, nickel may be taken up by the body through several routes, such as ingestion via food or water, absorption through the skin, and respiratory inhalation (122). Ni(II) compounds are human carcinogens, as epidemiological studies have decisively shown. Regular exposure to Ni-containing dusts and fumes increases the mortality rate of refinery workers due to respiratory tract cancers (1). Low water-soluble Ni compounds, such as crystalline nickel sulfide (NiS), nickel oxides (NO_x_)^18^, and crystalline nickel subsulfide (Ni_3_S_2_), are the most potential cancer causing nickel compounds in humans upon inhalation (123). Fossil fuel combustion is the main source of Ni compounds in the atmosphere. Direct leaching from rocks and sediments causes high level of divalent Ni and suspended insoluble particles in water (121). Long-term exposure to nickel compounds has been linked to nephrotoxicity, immunotoxicity, genotoxicity, lung toxicity, and carcinogenesis.

#### 2.5.1 Nickel toxicity

The toxicity of nickel compounds has been well recognized. It has been shown that cells tolerate nickel concentrations up to 1 mM for 24 hours without harm. Nickel causes hypoxia in tumors by displacing iron from the active site of deoxygenases, which is penta-coordinated, and because nickel is hexa-coordinated, there is no place for oxygen to bind (124). Nickel (Ni), a Group 10 transition metal essential for urease and hydrogenases at micromolar levels (dietary 0.1-1 μg/day), induces toxicity via industrial inhalation, dermal sensitization, and oral ingestion, with compound-specific effects tied to Ni^2+^ bioavailability-soluble salts (NiSO_4_) rapidly absorbed, insoluble Ni_3_S_2_/NiO phagocytosed for macrophage-mediated release. Nickel’s haptenation of skin proteins causes Type IV allergic contact dermatitis (13-18% prevalence, higher in females), resulting in eczematous rashes from jewelry/tools. Sensitized patients may develop systemic Ni allergy syndrome (SNAS) after ingestion (125, 126).

Respiratory toxicity is the most common occupational hazard. Soluble Ni causes rhinitis, asthma, and pneumonitis, while insoluble Ni causes fibrosis and pneumoconiosis. Inhaling Ni(CO)_4_ (LD50 0.3–1 ppm) causes delayed (12–48 hr) pulmonary oedema, myocarditis, seizures, and 38% mortality through lipid peroxidation. Acute oral NiSO_4_ (>1.6 g) produces nausea and vomiting, while persistent high urine Ni (>50 μg/g creatinine) affects renal and hepatic functions. Ni, a Group 1 carcinogen (subsulfide, oxide, or dust), epigenetically silences genes via H3K9me2 heterochromatin (127, 128).

#### 2.5.2 Epigenetic regulation in nickel carcinogenesis

Carcinogenesis induced by nickel is strongly linked to epigenetic dysregulation, which is essential for modifying gene expression without altering the DNA sequence. One key mechanism entails the suppression of iron- and 2-oxoglutarate-dependent dioxygenases by Ni^2+^ ions, leading to diminished histone demethylase activity and aberrant chromatin remodeling (129). Nickel exposure enhances histone methylation marks, specifically H3K9me2 and H3K4me3, resulting in chromatin condensation and transcriptional repression of tumor suppressor and DNA repair genes (130). Nickel compounds induce promoter hypermethylation of essential genes involved in DNA repair, apoptosis, and cell cycle control thereby facilitating malignant transformation and genomic instability (131). In addition to DNA methylation and histone modifications, nickel exposure markedly alters microRNA (miRNA) expression profiles that govern cell proliferation, death, inflammation, and metastasis. Nickel-mediated stabilization of HIF-1α simulates hypoxic conditions and activates oncogenic signaling pathways linked to angiogenesis and tumor growth (123). Moreover, prolonged nickel exposure produces an overabundance of ROS, leading to oxidative DNA damage and promoting epigenetic irregularities via inflammatory signaling pathways (132). Workers exposed to nickel commonly experience the onset of lung and nasal malignancies, which significantly correlates with the combination of epigenetic and oxidative modifications. Consequently, epigenetic control is a pivotal mechanism in nickel carcinogenesis and may yield intriguing diagnostic and therapeutic options for nickel-related malignancies.

### 2.6 Mercury

Mercury, the last element of the third transition series, is found in several physical and chemical forms in the Earth’s crust. At room temperature, it is a liquid. Among different mercury compounds (Hg^+1^, Hg^+2^), HgCl_2_ is the best known compound for its various applications, such as a fungicide in agriculture, as a precursor for other Hg-based compounds. Various forms of mercury, ranging from elemental mercury, which is liquid at room temperature and can slowly evaporate, releasing toxic vapours into the air, to inorganic mercury in the form of salt of sulfur or chlorine, are found in water, soil and dust. Exposure to these mercury species can occur through respiratory uptake, oral intake, or absorption through the skin, and is capable of causing significant adverse effects, including injury to the nervous and renal systems as well as other organ systems. Another form of mercury found in nature due to various transformations is organo mercury compounds like methylmercury, which is highly neurotoxic. The lipid-solubility of mercury makes it easily transported into the bloodstream. Its permeability through the biological membrane also affects the central nervous system and the kidneys (38, 133, 134).

#### 2.6.1 Mercury toxicity

Mercury has been found to interact preferentially with the -SH, -SeH, -COOH, -NH_2_, and -CONH_2_ groups of amino acids and can inhibit protein activity, increase ROS production, and thus interfere with the smooth functioning of various signaling pathways. For example, Glutathione (GSH), the most abundant and efficient antioxidant, acts as a ROS scavenger by catalysing the formation of water by reduction of H_2_O_2_ (or organic hydroperoxides) (135). The interaction of methylmercury (MeHg) form GS-MeHg complex with GSH which supress glutathione activity and induced oxidative stress and neurotoxicity. MeHg induces the formation of hydrogen peroxide and superoxide radicals, which lowers the inner mitochondrial membrane potential, and the electron transport chain is also inhibited. So, Oxidative stress thus produced causes mitochondrial damage resulting in more damage to astrocytes than to neurons (136, 137).

Similarly, another study investigated the role of methylmercury in the deregulation of Nrf2, a key transcription factor that regulates gene expression. Keap1 modulates Nrf2 signaling in response to oxidative stress induced by heavy metals such as Hg (137).

It has been found that the route of mercury poisoning involves the interaction of methylmercury (MeHg) with the cysteine protein in fish in an aquatic environment. The MeHg-cysteine complex can cross the blood-brain barrier easily and thus cause neurological disorders. Furthermore, the mercury intoxication is connected to cardiovascular, lung and renal diseases also. It has been reported that the cardiotoxicity caused by mercury is associated with myocardial infarction, hypertension, atherosclerosis, etc. (133). The most important epigenetic modifications induced by mercury intoxication are DNA methylation and histone post-translational modifications in kidney tissue.

#### 2.6.2 Epigenetic regulation in mercury carcinogenesis

**Figure 3:**
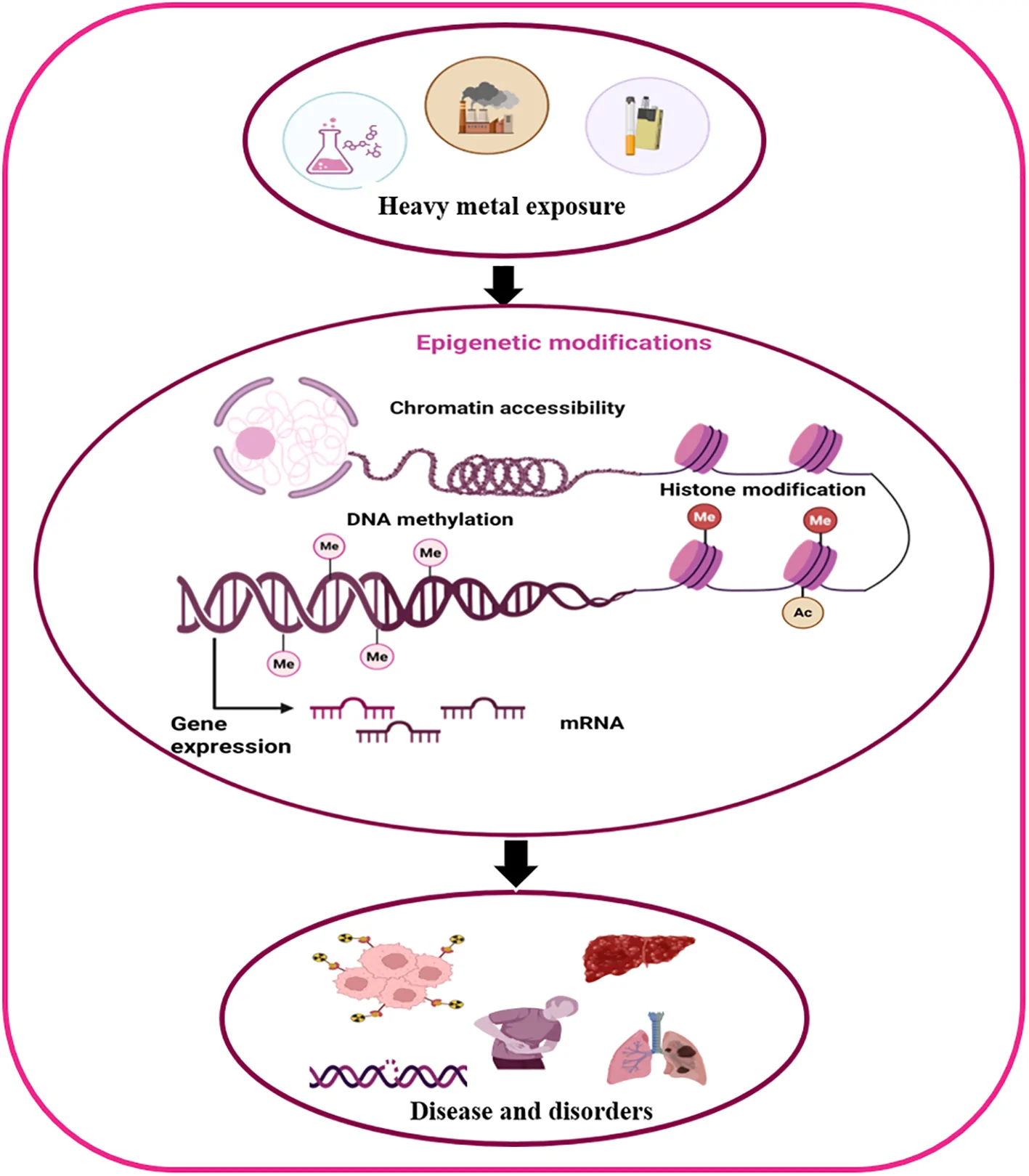
Epigenetic modifications due to metal carcinogenesis.

As discussed earlier, the epigenetic pathway has been identified as one of the most important roadmaps for studying the mechanisms of metal-induced carcinogenesis. It refers to a change in gene expression without a change in the sequence of DNA due to DNA methylation, microRNA activity, and histone modifications (138) (**Figure 3**). Literature is rich in reports that demonstrate mercury exposure triggers epigenetic dysregulation, especially in the kidneys, immune system, nervous system, etc. Emerging evidence has shown that exposure to MeHg causes transgenerational neurotoxicity through epigenetic alterations in critical genes, such as the neurotrophin BDNF. Also, MeHg exposure has been found to alter chromatin structure by altering the expression of upstream regulators, such as HDACs and DNMTs, thereby regulating DNA methylation as well as histone modifications (139).

Another piece of evidence of epigenetic alteration by Hg exposure has reported that cervical miRNA appears to be a novel indicator of maternal mercury exposure in pregnancy, whereby Hg-triggered epigenetic changes are implicated in diverse pathologies, including decreased cerebellar size in neonates, detrimental behavioral effects, and atherosclerosis. Different studies on various mechanisms of gene function alteration have found that DNA methylation and histone post-translational modifications are the predominant epigenetic alterations in Hg (140).

## 3 Molecular mechanisms underlying metal-induced initiation and progression of cancer

Cancer arises when normal cells accumulate molecular alterations that permit unchecked growth, resistance to programmed cell death, and invasion of adjacent tissue. Environmental carcinogens drive this process by either directly damaging DNA or disrupting gene regulation, and heavy metals are particularly notable because they often act through both routes simultaneously. Certain metals produce only limited mutagenic damage but exert powerful effects on the epigenome, whereas others are potent genotoxic agents in their own right; regardless of the specific route, the result is often a population of transformed cells with heightened survival and proliferative potential (7, 18).

**Figure 4:**
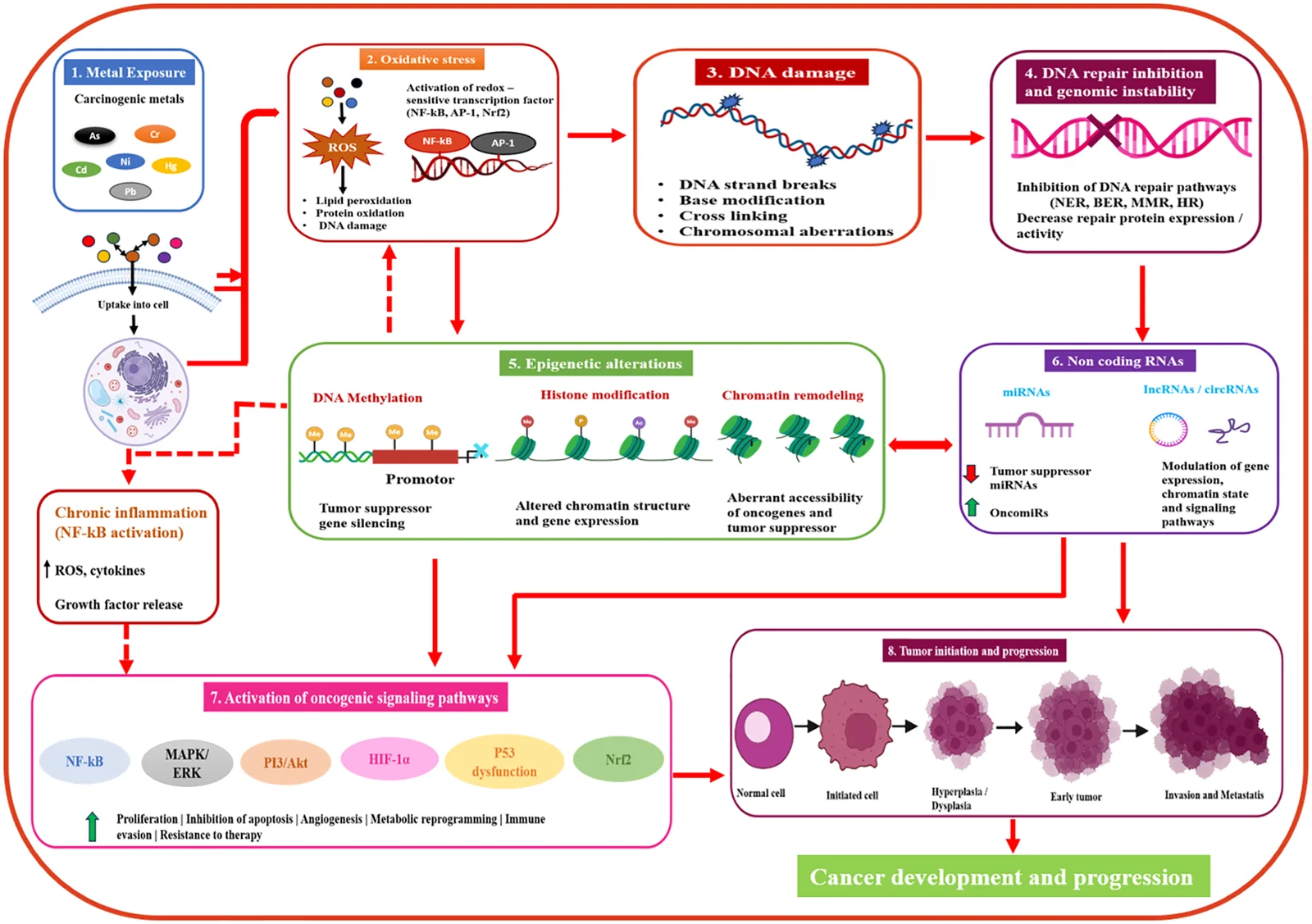
Mechanism of metal carcinogenesis.

An all-inclusive understanding of the mechanisms behind the development of metal-induced cancer can provide valuable insights for impending cancer therapeutics. A growing body of reports indicates that metal-induced carcinogenesis cannot be attributed to any single lesion or pathway. Instead, it emerges from an interconnected web involving oxidative stress, DNA damage and its repair, and dysregulated signal transduction (9, 141). More recently, attention has turned to epigenetic contributions to this process, with changes in DNA methylation, histone modification, and non-coding RNA expression now recognized as important drivers of metal-induced cellular transformation (16). Although these mechanisms are frequently discussed as distinct categories in the literature, they are biologically intertwined: oxidative stress can both directly damage DNA and modify enzymes that control chromatin structure, while epigenetic changes can lock in aberrant patterns of gene expression and help damaged cells evade elimination. Appreciating this cross-talk is essential to understanding how acute molecular injury gradually develops into long-term cancer risk (7, 18, 142) (**Figure 4**).

### 3.1 Oxidative stress as the initiating event

Oxidative stress is typically among the first cellular responses triggered by metal exposure and serves as the central hub linking downstream molecular changes. Redox-active metals, such as chromium and arsenic, generate reactive oxygen species (ROS) directly through intracellular redox cycling. Metals that lack this redox activity, including cadmium and nickel, raise ROS levels indirectly by disrupting mitochondrial electron transport, depleting cellular glutathione stores, and suppressing antioxidant enzymes such as superoxide dismutase, catalase, and glutathione peroxidase (64, 143, 144). When ROS accumulation exceeds the capacity of the cell’s antioxidant systems, lipids, proteins, and nucleic acids are all subject to oxidative damage. This excess ROS also activates redox-sensitive transcription factors, such as NF-κB and AP-1, driving chronic inflammation and sustained cytokine and chemokine release. These inflammatory mediators, in turn, generate additional ROS, forming a self-reinforcing loop of oxidative injury and inflammatory signaling that creates conditions favorable to malignant transformation (145, 146).

### 3.2 From oxidative damage to genomic instability

This sustained oxidative environment feeds directly into genomic instability, producing a broad range of DNA lesions. Excessive ROS oxidizes DNA bases to form lesions such as 8-hydroxy-2′-deoxyguanosine (8-OHdG), along with strand breaks, DNA-protein cross-links, and chromosomal aberrations (62, 147). Chromium (VI) is especially damaging because its intracellular reduction generates highly reactive intermediates capable of forming DNA adducts and interstrand cross-links, while arsenic contributes to genomic instability largely by impairing DNA repair machinery and disrupting mitosis rather than reacting with DNA directly (128). Cadmium and nickel add to this mutagenic burden by interfering with zinc-dependent DNA repair proteins, allowing oxidative lesions to accumulate unchecked. At the same time, these metals impair several major repair pathways — nucleotide excision repair (NER), base excision repair (BER), mismatch repair (MMR), and homologous recombination — further eroding the cell’s capacity to preserve genomic integrity (148, 149). As unrepaired damage accumulates, mutations arise in oncogenes and tumor suppressor genes, setting the stage for malignant transformation and clonal expansion (150).

### 3.3 Epigenetic reprogramming locks in genetic damage

The genetic changes triggered by oxidative stress are reinforced and stabilized through extensive epigenetic reprogramming. Heavy metals interfere with DNA methyltransferase (DNMT) activity and disturb one-carbon metabolism, and exposed cells commonly display a combination of global DNA hypomethylation alongside promoter-specific hypermethylation at tumor suppressor loci. Global hypomethylation contributes to chromosomal instability and can reactivate oncogenic elements, while hypermethylation at specific promotes silences genes governing DNA repair, apoptosis, and cell-cycle control, such as CDKN2A, MLH1, and RASSF1A (151–153). Notably, this relationship runs in both directions: oxidative stress can itself reshape DNA methylation patterns, and the resulting methylation abnormalities further suppress antioxidant and DNA repair gene expression, perpetuating cellular dysfunction.

Alongside changes in DNA methylation, carcinogenic metals also alter histone modifications and broader chromatin architecture, with downstream effects on gene expression and tumor development. Metal exposure disrupts histone-modifying enzymes, including histone acetyltransferases, histone deacetylases, and histone methyltransferases - leading to abnormal chromatin states (18, 154, 155). Nickel exposure favors repressive marks such as H3K9me2 and H3K27me3, whereas chromium and cadmium instead alter patterns of histone acetylation and methylation; in both cases, the outcome is silencing of tumor suppressor genes coupled with activation of oncogenic pathways (16, 130, 153). Because these chromatin changes are reversible, they represent an appealing therapeutic target, even as they continue to drive proliferation, inflammation, angiogenesis, and resistance to apoptosis in exposed cells (151, 156).

### 3.4 Non-coding RNAs as integrators of the response

Non-coding RNAs (ncRNAs) have emerged as important links connecting oxidative stress and epigenetic dysregulation to metal-induced carcinogenesis. Exposure to carcinogenic metals alters the expression of microRNAs (miRNAs), long non-coding RNAs (lncRNAs), and circular RNAs (circRNAs), with downstream consequences for gene expression and cell signaling (157–159). As one example, miR-21 upregulation activates the PI3K/Akt pathway by suppressing PTEN, while loss of miR-200 expression promotes epithelial-mesenchymal transition (EMT) and metastatic spread (160). LncRNAs including HOTAIR and MALAT1, together with various circRNAs, additionally influence chromatin remodeling and act as competing endogenous RNAs (ceRNAs), reinforcing oncogenic signaling networks and supporting tumor progression (16, 161, 162). Taken together, these ncRNAs serve as a molecular bridge that connects oxidative stress, epigenetic alteration, and aberrant signaling in the progression of metal-induced cancer.

### 3.5 An integrated view of metal carcinogenesis

Ultimately, oxidative stress, DNA damage, impaired repair capacity, epigenetic alteration, and ncRNA dysregulation all converge on a shared set of signaling pathways, including NF-κB, MAPK, PI3K/Akt, HIF-1α, and p53. Sustained activation of these pathways supports uncontrolled cell proliferation, resistance to apoptosis, angiogenesis, chronic inflammation, immune evasion, metabolic reprogramming, and metastasis (146, 150). Rather than acting as separate, independent events, these mechanisms reinforce one another through positive feedback loops, driving the stepwise progression from normal cells through precancerous lesions to invasive and metastatic disease (150). Viewed this way, metal-induced carcinogenesis is best understood not as a collection of isolated molecular events but as an integrated network of genetic and epigenetic changes, a framework that opens multiple avenues for biomarker discovery and targeted therapeutic development (150, 156).

## 4 Therapeutic action

**Figure 5:**
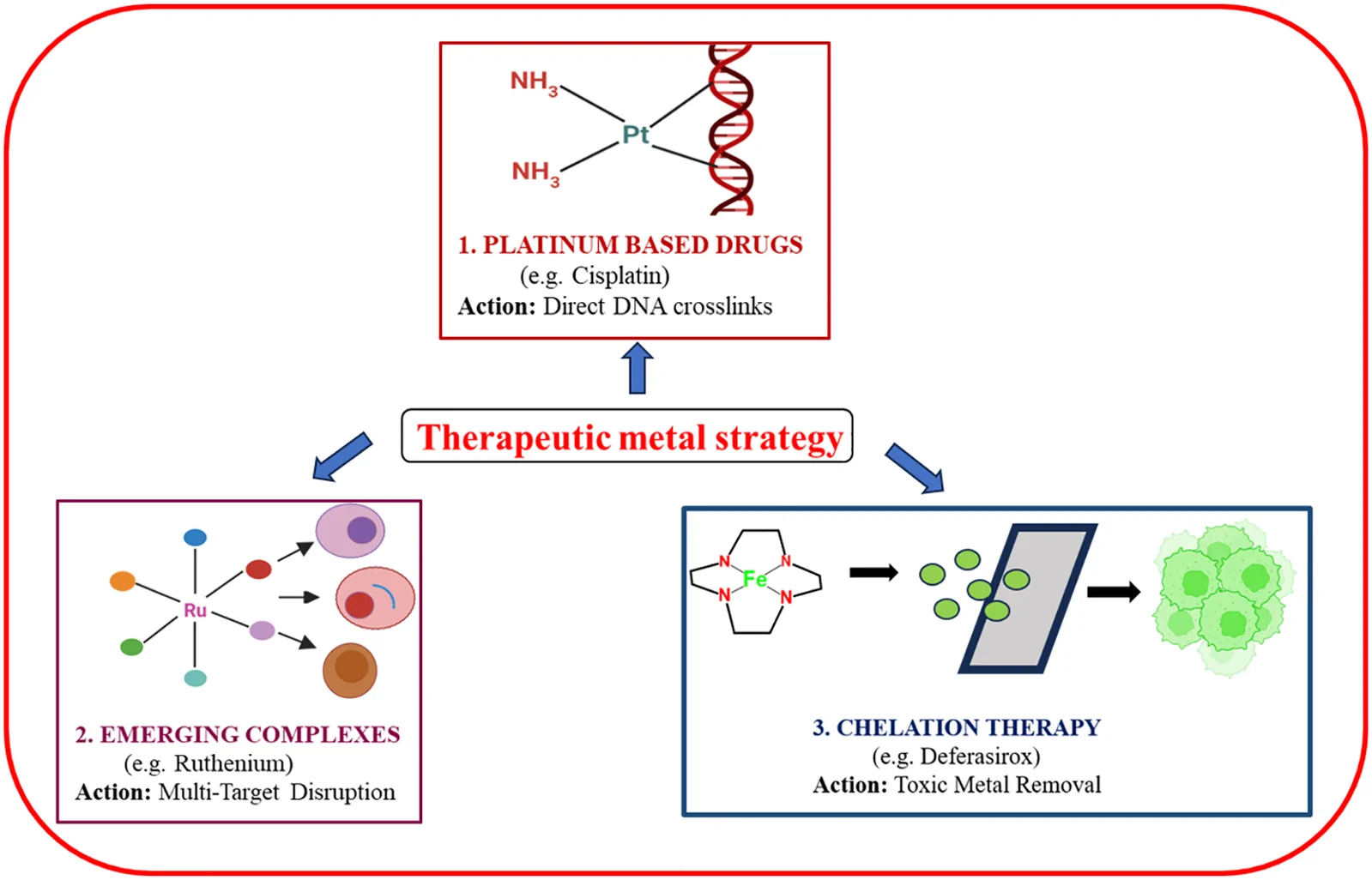
Therapeutic action of metal drugs.

Heavy metals (e.g., Cd, As, and Cr) are recognized as environmental carcinogens. They infiltrate living organisms *via* air, water, and food chains, inducing cancer by generating ROS, creating DNA adducts, and disrupting critical signaling pathways. The identical redox and coordination chemistry that renders them lethal also renders them beneficial for therapeutic applications. For example, Cisplatin (*cis*-diamminedichloroplatinum), a platinum-based drug, creates intrastrand cross-links with DNA, simulating damage that selectively targets and eliminates fast proliferating tumor cells. This metal duality clarifies the mechanisms of carcinogenesis and advances next-generation chemodynamic therapies and metal-organic frameworks (MOFs) for precision oncology. When metals enter aqueous biological environments, they can undergo oxidation to form cationic species that exhibit strong electrostatic affinity for anionic sites on biomolecules, including phosphate groups on nucleic acids and carboxylate residues on proteins. The coordination chemistry of these metal centers governs local charge distribution, thereby modulating the electrostatic surface potential and conformational stability of associated macromolecules. Metal ions with elevated electron affinities and Lewis acidity polarise coordinated water molecules and substrate functional groups, lowering activation barriers for hydrolytic cleavage of phosphodiester and peptide bonds (163). The properties of metals have recently attracted significant interest in the potential therapeutic applications of medical inorganic chemistry for the development of anti-cancer medication. In this part of article, we briefly discuss the therapeutic actions of some important drugs used to treat various diseases, with a main focus on cancer (**Figure 5**).

### 4.1 Platinum-based drugs

The discovery of cisplatin in the 1960s marked a significant advancement in cancer treatment. The success of cisplatin therapy in cancer treatment has prompted substantial research in bioinorganic chemistry (164). Carboplatin, oxaliplatin, nedaplatin, lobaplatin, heptaplatin, and phenanthriplatin are some metallodrugs that have undergone considerable research and are clinically approved in various countries. They are used to treat a variety of cancers and are key components of combination therapies such as immunotherapy (165).

Platinum-based drugs primarily exert their anticancer effects by binding to DNA. This binding stops DNA replication and triggers programmed cell death. Recent studies focus on immunogenic cell death (ICD) and customized delivery methods that improve effectiveness and reduce resistance (166). These drugs enter cells via transporters such as CTR1 and OCTs. Once inside, they react to form the [Pt(NH_3_)_2_(Cl)(H_2_O)]^+^ species. This species binds to the N7 position of guanine in DNA, leading to crosslinks between and within DNA strands. This process halts replication and transcription, initiates apoptosis through p53, and causes G2/M arrest. Oxaliplatin is the only drug that induces immunogenic changes via endoplasmic reticulum stress. Other unintended effects include mitochondrial damage, the production of ROS, and proteasome inhibition (167, 168). Recent studies show that liposomal cisplatin, such as in lung cancer trials, reduces accumulation in healthy tissues through tailored delivery. Pt (IV) prodrugs can be activated by light or used with photodynamic therapy (PDT) to release active Pt (II) into tumors. This results in damage to ROS and DNA. Phenanthriplatin disrupts transcription via bulky ligands, effectively targeting cells that are resistant to cisplatin. HER2-targeted platinum conjugates improve specificity in breast cancer treatment (168, 169).

### 4.2 Emerging metal complexes

For many years, the usage of metal-based complexes in cancer therapy has faced several problems, particularly with Pt complexes and Pt-derived drugs in clinical settings. While platinum-based chemotherapy is generally effective, it is important to mention its limitations, including drug resistance, a narrow range of effectiveness, and the risk of worsening side effects. Researchers have assessed other metal-based complexes with toxic properties, for example those containing Ru, Au, and Fe, to overcome these challenges. The ruthenium complexes designed for this use exhibit improved tolerability, characterized by fewer and milder side effects (170).

Ru-containing drugs like KP1019 (now NKP-1339), NAMI-A, and RAPTA-T were initially created as “platinum analogues” that could bind to DNA. However, they have better properties, such as lower toxicity to healthy cells and effectiveness against metastases and tumors resistant to other treatments. Ru is delivered as inactive Ru(III) prodrugs that become active when reduced in the hypoxic areas of tumors. This approach allows targeted activation while sparing other cells. The drugs also disrupt proteins, cause stress in the endoplasmic reticulum, affect mitochondria, and interfere with migratory pathways (p38 MAPK), limiting cancer spread like traffic enforcement (171, 172) NAMI-A effectively prevents metastases by changing how cells stick together. On the other hand, NKP-1339 activates the unfolded protein response (UPR) to induce cell death, even in areas where platinum struggles to enter. The EPR effect, which involves leaky tumor blood vessels, requires lower doses (173).

### 4.3 Chelation therapy

Chelation treatment is a targeted approach to inhibit cancer metastasis by eliminating excess toxic metals that facilitate tumor proliferation. It employs agents such as EDTA, deferoxamine (DFO), or deferasirox to sequester free metal ions, including lead, cadmium, arsenic, and iron (174, 175). These chemicals form stable complexes that are excreted in urine. This reduces the likelihood that ROS will form and cause DNA damage. In cancer situations, this disrupts metal-dependent processes, including Fenton reactions (iron-driven reactive oxygen species), which facilitate mutations, inflammation, and tumor proliferation, particularly in breast or colorectal malignancies characterized by excessive iron levels. The dual advantages comprise direct cytotoxicity against cancer cells that rely on metals for rapid proliferation, as well as synergistic effects with chemotherapy and radiotherapy by enhancing oxidative stress and inhibiting DNA repair mechanisms. Iron chelation therapy has attracted significant interest over the years as a potential cancer treatment, leveraging tumor-induced increased iron demand. It functions by selectively extracting iron from tumor cells, thereby altering iron storage within those cells and inhibiting iron-dependent oncogenic pathways. This differs from traditional chelation therapies, which solely address systemic iron overload (176, 177).

Platinum-based drugs remain the benchmark for directly targeting cancer cell DNA, yet ruthenium compounds are emerging as promising, less harsh alternatives with broader effects. Iron chelation therapy takes a different path altogether, depriving tumors of the essential iron they need to thrive. Together, these approaches from precise DNA strikes (platinum), to versatile disruptions (ruthenium), to strategic depletion (iron removal) highlight the shift in metal-based treatments toward more nuanced strategies. This evolution shows that metals play two roles in cancer development. Excess heavy metals, such as cadmium or arsenic, damage DNA, generate reactive oxygen species, and activate oncogenes, leading to tumor formation. These therapies effectively address this issue by trapping, redirecting, or removing metal-induced disorders to stop progression and restore balance.

## 5 Conclusion

Heavy metals are the most harmful environmental pollutants to human health. Heavy metals that people inhale or consume through food and water can accumulate in their bodies, causing long-term health issues. These health effects may include inflammation and damage to vital organs, which, when prolonged, can increase the risk of cancer. Heavy metal carcinogenesis is increasingly recognized as a dynamic and complex process in which epigenetic instability serves as a critical mechanism linking environmental exposure to malignant transformation. Cadmium, arsenic, nickel, and chromium are highly hazardous metals that cause oxidative stress and chromosomal instability. They also alter the epigenome through changes in DNA methylation, histone modifications and non-coding RNA expression. These changes disrupt gene regulatory networks, repressing tumor suppressor genes and activating oncogenic pathways, hence enabling cancer genesis, progression, and persistence. It is important to note that epigenetic alterations differ from genetic mutations in their durability and reversibility, which creates a possibility for therapeutic intervention that is critical. Recent developments in high-throughput epigenomic sequencing have made the identification of exposure-specific epigenetic patterns easier. These patterns have presented potential biomarkers for early detection, risk assessment, and prognosis. Furthermore, epigenetic treatments like DNA methyltransferase inhibitors, histone deacetylase inhibitors, and emerging RNA-based approaches have significant potential to reverse epigenetic modifications induced by metals and restore normal physiological function in cells. The application of nanotechnology in conjunction with individualized medication delivery systems has the potential to generate therapies that are even more effective and more personalized. A comprehensive understanding of the interaction between heavy metal exposure and epigenetic regulation not only advances our understanding of cancer biology but also enables the development of novel, mechanism-driven therapeutic approaches. This, in turn, leads to improved cancer prevention, diagnosis, and individualized treatment of cancers that are caused by environmental factors.

## 6 Future perspective

Future research in heavy metal carcinogenesis and epigenetic regulation will increasingly focus on gaining a thorough and mechanistic understanding of how environmental exposures modify the epigenome, causing cancer genesis and progression. High-throughput epigenomic technologies, such as EWAS and next-generation sequencing, can identify metal-specific epigenetic signatures as sensitive biomarkers for early detection, exposure assessment, and prognosis (18, 166). Future studies are expected to explore the effects of prolonged, low-level cumulative exposures, which better approximate actual environmental conditions, on epigenetic regulators including DNA methylation, histone modifications, and non-coding RNA networks (178). Furthermore, rising data showing transgenerational epigenetic inheritance emphasizes the necessity for long-term cohort research to better understand how early-life exposures influence disease risk across generations (179). Importantly, the reversibility of epigenetic changes opens up intriguing therapeutic prospects, such as the use of DNA methyltransferase and histone deacetylase inhibitors, as well as upcoming CRISPR-based epigenome editing and RNA-targeted medicines (19). The integration of nanotechnology-based medication delivery systems with artificial intelligence-driven multi-omics analysis is expected to enhance precision medicine in this field. These breakthroughs will help to translate molecular knowledge into viable preventive, diagnostic, and therapeutic measures, thereby lowering the burden of heavy metal-induced malignancies.

## Glossary

G2/M: gap 2/mitosis
CTR1: copper transporter 1
OCTs: organic cation transporters
ICD: immunogenic cell death
MOFs: metal-organic frameworks
HDACs: histone deacetylase
DNMTs: DNA methylation
BDNF: brain-derived neurotrophic factor
NRF2: nuclear factor erythroid 2-related factor
ECH: enoyl-CoA hydratase
KEAP1: kelch-like ech-associated protein 1
GSH: glutathione
HIF-1α: hypoxia-inducible factor 1-alpha
IARC: international agency for research on cancer
ncRNA: non-coded ribose nucleic acid
ROS: reactive oxygen species
WHO: world health organization
SH: sulfhydryl
ATP: adenisine tri-phosphate
SAM: s-adenosylmethionine
miRNAs: microRNAs
lncRNAs: long non-coding RNAs
MAPK: mitogen-activated protein kinase
p^38^MAPK: p^38^ mitogen-activated protein kinase
NF-κB: nuclear factor kappa-light-chain-enhancer of activated B cell
EWAS: epigenome-wide association study
EDTA: ethylenediaminetetraacetic acid
DFO: deferoxamine
EPR: enhanced permeability and retention
UPR: unfolded protein response
NKP-1339: sodium trans-[tetrachloridobis(1H-indazole)ruthenate(III)]
NAMI-A: new anticancer metastasis inhibitor
RAPTA-T: ruthenium-arene-pta toluene
KP1019: indazolium trans-[tetrachlorobis(1H-indazole)ruthenate(III)]
PDT: photodynamic therapy
KRAS: kirsten rat sarcoma viral oncogene homolog
RRAGC: ras-related gtp binding c
HAT: hepatic artery thrombosis
H19 ICR: h19 imprinting control region
hESCs: human embryonic stem cells
LINE-1: long interspersed element-1
BLLs: blood lead levels
TNF-α: tumor necrosis factor-alpha
IL-6: interleukin-6
PDCD4: programmed cell death 4
Cdln1c: cyclin-dependent kinase inhibitor 1c
H4K12ac: aetylation of histone H4 at the 12th lysine residue
PI3K: phosphoinositide 3-kinase
AKT: protein kinase b
DKK1: dickkopf-1
8-OHdG: 8-hydroxy-2′-deoxyguanosine
NER: nucleotide excision repair
MMR: mismatch repair
BER: base excision repair
EMT: epithelial-mesenchymal transition
HOTAIR: hox transcript antisense RNA
MALAT1: metastasis-associated lung adenocarcinoma transcript 1
CDKN2A: cyclin-dependent kinase inhibitor 2A
MLH1: MutL protein homolog 1
